# Nanomaterial-induced pyroptosis: a cell type-specific perspective

**DOI:** 10.3389/fcell.2023.1322305

**Published:** 2024-01-09

**Authors:** Zhiyong Wang, Min Wang, Xuan Zeng, Xupeng Yue, Pei Wei

**Affiliations:** ^1^ Department of Immunology, Zhuhai Campus of Zunyi Medical University, Zhuhai, China; ^2^ Department of Pharmaceutics, Zhuhai Campus of Zunyi Medical University, Zhuhai, China; ^3^ Department of Pharmaceutics, Guangdong Provincial People’s Hospital Zhuhai Hospital, Zhuhai, China; ^4^ College of Bioengineering, Zhuhai Campus of Zunyi Medical University, Zhuhai, China

**Keywords:** nanomaterials, pyroptosis, therapeutic applications, toxicology, non-tumor cells

## Abstract

This review presents the advancements in nanomaterial (NM)-induced pyroptosis in specific types of cells. We elucidate the relevance of pyroptosis and delineate its mechanisms and classifications. We also retrospectively analyze pyroptosis induced by various NMs in a broad spectrum of non-tumorous cellular environments to highlight the multifunctionality of NMs in modulating cell death pathways. We identify key knowledge gaps in current research and propose potential areas for future exploration. This review emphasizes the need to focus on less-studied areas, including the pathways and mechanisms of NM-triggered pyroptosis in non-tumor-specific cell types, the interplay between biological and environmental factors, and the interactions between NMs and cells. This review aims to encourage further investigations into the complex interplay between NMs and pyroptosis, thereby providing a basis for developing safer and more effective nanomedical therapeutic applications.

## Introduction

Nanomaterials (NMs) have emerged as a promising avenue in biomedicine because of their unique physical and chemical properties. Their applications cover diverse areas, including drug delivery, bioimaging, and disease treatment (Sasidharan and Monteiro-Riviere, 2015). However, with the increasing use of NMs, concerns over their potential effects on biological systems are escalating. A particular focus of current research is NM-induced pyroptosis.

Pyroptosis is a form of regulated cell death dependent on the formation of plasma membrane pores by the gasdermin (GSDM) protein family (Shi et al., 2017). It fundamentally represents an adaptive response of cells to external stimuli. It plays a crucial role in host defense against microbes, cytokine secretion, inflammation, and tumor immunity (Wei et al., 2022). Recent advancements in nanotechnology have led to the widespread application of NMs in the activation and improvement of pyroptosis to improve the efficacy of cancer treatments (Wu et al., 2021; Chen et al., 2022). While nanomedicine-induced pyroptosis has emerged as an effective strategy for tumor therapy, pyroptosis has advantages and disadvantages. Its effect on the human body can vary dramatically across different genetic backgrounds, tissues, and disease stages. NMs or their degradation products may also trigger pyroptotic toxicity in normal cells/tissues although they can induce pyroptosis to combat tumors. Notably, NM-induced pyroptosis in immune cells may disrupt the body’s immune homeostasis; consequently, it affects immune defense capabilities and even causes irreversible tissue damage (Wei et al., 2022). Therefore, minimizing nonspecific damage is a critical issue that must be addressed for the clinical application of NMs. The extent of NM-induced pyroptosis primarily depends on factors such as the characteristics of NMs, dosage, exposure time, and the nature of the interaction between NMs and cells (Andón and Fadeel, 2013). Therefore, an in-depth understanding of these factors can provide insights into the varying degrees of pyroptotic responses and a basis for developing safer and more effective medical applications of NMs.

While studies have described the prospects for tumor treatment via NM-induced pyroptosis, our understanding of the effect of such materials on nontumor cells, particularly within the context of immune cells, has a notable gap. In this review, we aim to provide a comprehensive overview of the current research progress on NM-induced pyroptosis in specific cell types, elucidate existing controversies, and identify gaps in our understanding. Thus, our goal is to offer readers an enhanced understanding of this complex yet critical field and propose potential directions for future research.

## Definition and classification of pyroptosis

### Overview of pyroptosis

Pyroptosis is a regulated form of cell death that relies on the GSDM protein family to form pores in the plasma membrane (Shi et al., 2017). In this process, holes form in the cell membrane, and the cell continuously enlarges until the membrane ruptures; consequently, cellular contents, such as certain cytokines, are released. Since pyroptosis was first observed in macrophages (Gery et al., 1981), our understanding of this form of cell death has undergone remarkable developments. Pyroptosis is mediated by GSDM family molecules rather than inflammasomes or caspases, suggesting that GSDMs can trigger pyroptosis in various cell types through mechanisms that do not depend on inflammasomes and caspases. In addition to the action of various proteases, the activation of GSDM molecules is tightly regulated by post-translational modifications such as succinylation (Humphries et al., 2020), palmitoylation (Balasubramanian et al., 2023; Du et al., 2023), ubiquitination (Shi et al., 2022), and oxidation (Devant et al., 2023). Their activation is independent of the occurrence of pyroptosis because of the dynamic nature of the process involving GSDM-mediated pore formation, plasma membrane rupture, and intracellular cytokine release, which are regulated by multiple factors. Additionally, pyroptosis interacts with other cell death pathways, including the potential for a switch between cell death modes.

The GSDM family serves as executioner molecules of pyroptosis, and its pore-forming function is a prerequisite for pyroptosis (Shi et al., 2015; Shi et al., 2017). It comprises six members in the human body: GSDMA, GSDMB, GSDMC, GSDMD, GSDME, and DFNB59 (Ding et al., 2016; Broz et al., 2020). The structures of all GSDMs except DFNB59 include a cytotoxic N-terminal pore-forming domain (PFD) and a C-terminal repressor domain (RD) (Broz et al., 2020). Under normal circumstances, the PFD and RD aggregate together, inhibiting the pore-forming function of GSDM (Liu et al., 2019; Broz et al., 2020). Once activated by specific signals, GSDM molecules are cleaved by active caspases or granzymes, thereby separating the N- and C-termini. The cleaved N-terminal domain can insert into the cell membrane and assemble to form large oligomeric pores. GSDM pores can disrupt the integrity of the cell membrane; consequently, inflammatory cell death is triggered, and through this process, cellular contents, including inflammatory cytokines, are released into the extracellular space. During pyroptosis, GSDMs must be cleaved by upstream active caspases or other proteases for activation. Therefore, pyroptosis can be classified into four types based on different proteases: classical inflammasome pathway (caspase-1 mediated), nonclassical inflammasome pathway (caspase-4/5/11 mediated), apoptosis-related caspase (caspase-3/6/7/8)-mediated pathway, and other proteases-mediated pathway.

### Classical inflammasome pathway (caspase-1 mediated)

The classical pyroptosis pathway is mediated by the inflammasome assembly, which is activated by recognizing pathogen-associated molecular patterns (PAMPs) and danger-associated molecular patterns (DAMPs); this process is accompanied by the cleavage of GSDMD and the release of IL-1β and IL-18 (Shi et al., 2014; Liston and Masters, 2017). Generally, the inflammasome consists of intracellular pattern recognition receptors (PRRs), apoptosis-associated speck-like proteins containing a caspase recruitment domain (ASC), and inflammatory caspases (Lamkanfi and Dixit, 2014; Yu et al., 2021). Common PRRs, such as nucleotide-binding oligomerization domain-like receptors (NLRs, including NLRP1, NLRP3, and NLRC4), AIM2, and pyrin, have been extensively studied (Rathinam et al., 2010; He et al., 2016; Rathinam and Fitzgerald, 2016). They recognize various stimuli, such as toxins, pathogens, and metabolites, and activate downstream signaling pathways. The inflammasome adapter ASC, which has PYD and CARD domains, can recruit pro-caspase-1 (Hornung et al., 2009; Fernandes-Alnemri et al., 2010; Matyszewski et al., 2018). After the inflammasome assembly, caspase-1 is activated, cleaving GSDMD and the IL-1β and IL-18 precursors; consequently, pyroptosis occurs, and inflammatory cytokines are released. During this process, a cascade of intracellular events may take place. For instance, Miao et al. discovered that GSDMD-NT can directly damage the mitochondria during pyroptosis; as a result, 3ʹ–5ʹ exoribonucleases are released into the cytoplasm. Thus, mRNA is extensively degraded, thereby exacerbating cell pyroptosis and amplifying subsequent inflammatory responses (Miao et al., 2023). However, the resulting post-inflammasome activation can vary; for example, it can lead to pyroptosis or may cause cytokine release without cell death (Carty et al., 2019). Although the exact mechanisms accounting for these differences are unclear, they may involve the regulation of cell membrane rupture and repair during pyroptosis (Kayagaki et al., 2021; Chai et al., 2022).

### Nonclassical inflammasome pathway (caspase-4/5/11 mediated)

In non-classical pyroptosis pathways, the upstream sensing complexes of human caspase-4/5 (mouse caspase-11) are absent; as such, these caspases can bind directly to signaling inducers such as bacterial lipopolysaccharides (LPS) through their N-terminal CARD. Consequently, they become activated (Shi et al., 2014). The activated caspase-4/5/11 can cleave GSDMD into N-GSDMD, which then oligomerizes and translocates to the cell membrane; as a result, membrane pores are formed, and pyroptosis is induced (Aglietti et al., 2016). While caspases-4/5/11 can initiate pyroptosis via GSDMD, their role in the processing of pro-inflammatory cytokines is relatively inefficient. Notably, these caspases do not effectively cleave pro-IL-1β (Exconde et al., 2023). Thus, the optimal maturation and release of IL-1β and IL-18 typically require NLRP3 inflammasome activation, followed by caspase-1 action. NLRP3 activation involves a cascade of mitochondrial reactive oxygen species production, ATP release, and K^+^ efflux (Ruhl and Broz, 2015; Weindel et al., 2022). These insights highlight a complex regulatory network where non-classical inflammasome modulate canonical pathways to fine-tune host immune responses (Baker et al., 2015; Ruhl and Broz, 2015; Schmid-Burgk et al., 2015).

### Apoptosis-related caspase (caspase-3/6/7/8)-mediated pathway

In addition to caspase-1/4/5/11, apoptosis-related caspases such as caspase-8, caspase-3, caspase-7, and caspase-6 contribute to pyroptosis. Under certain conditions, such as *Yersinia* infection or specific kinase inhibition, macrophages can utilize caspase-8 as an alternative to caspase-1 for the cleavage of GSDMD, thereby inducing pyroptosis (Orning et al., 2018; Sarhan et al., 2018). Conversely, upon activation, caspase-3 and -7 can cleave GSDMD at Asp87 (Asp88 in mice); as a result, it becomes inactivated, and its ability to localize to membranes is inhibited, effectively suppressing pyroptosis. Additionally, tumor cell pyroptosis induced by chemotherapeutic drugs or macrophage-derived tumor necrosis factor α (TNF-α) can occur through the caspase-8/GSDMD/GSDME/GSDMC pathway (Hou et al., 2020). Unlike GSDMD, GSDME is directly situated within the apoptotic pathway, and its expression levels dictate the switch between apoptosis and pyroptosis to some extent. This switch, induced by chemotherapeutic drugs or cytokines such as TNF-α, primarily depends on GSDME expression levels (Rogers et al., 2017; Wang et al., 2017). In GSDMD-deficient macrophages, GSDME activation by caspase-3 not only induces pyroptosis but also facilitates cytokine secretion at various stages of this cell death process (Zhou and Abbott, 2021). In macrophages lacking caspase-1/11, NLRP3 can activate caspase-3/8, which then cleaves GSDME, triggering a form of incomplete pyroptosis (Aizawa et al., 2020). This process does not involve IL-1β release; instead, it involves IL-1α secretion. Another entity participating in non-inflammatory caspase-mediated pyroptosis is caspase-6. It enhances the interaction between receptor-interacting protein kinase 3 (RIPK3) and Z-DNA-binding protein 1 (ZBP1); thus, it activates the NLRP3/caspase-1 signaling pathway (Zheng et al., 2020).

### Pyroptosis mediated by other proteases

Granzymes, a family of serine proteases, have been recognized as cell death mediators (Bots and Medema, 2006; Voskoboinik et al., 2015). They can modulate inflammation by directly or indirectly inducing pyroptosis. Granzyme A (GZMA), derived from cytotoxic T lymphocytes, cleaves GSDMB. This interaction creates pores in the membrane and induces pyroptosis in GSDMB-expressing cancer cells (Zhou et al., 2020). However, this process is contingent on the expression of GSDMB, which is absent in some human tissues and mice. In parallel with GZMA, granzyme B (GZMB) derived from natural killer cells can induce pyroptosis. GZMB directly cleaves GSDME at the same site as caspase-3; consequently, its effector N-terminal is released, and the cell membrane is perforated (Zhang et al., 2020). This process can occur in two distinct routes: directly through GSDME cleavage or indirectly through caspase-3 activation. This process may amplify inflammatory responses in a tumor microenvironment, recruiting more immune cells for antitumor immunity. Furthermore, chimeric antigen receptor T cells stimulate caspase-3, which can cleave GSDME, causing pyroptosis in target cells (Liu et al., 2020). Importantly, this process can occur regardless of the presence or absence of caspase-3; therefore, granzymes can induce pyroptosis via direct and indirect pathways. The internalization of GSDME- and GSDMB-activated granzymes requires perforin derived from lymphocytes (Zhang et al., 2020; Zhou et al., 2020). Thus, GSDME- and GSDMB-mediated pyroptosis is considered a primary terminal effector of the perforin-granzyme cytotoxic pathway. In neutrophils, GSDMD can be cleaved by elastase and cathepsin G in azurophilic granules; as a result, pyroptosis is induced, and IL-1β is secreted (Burgener et al., 2019). In human and mouse keratinocytes, streptococcal pyrogenic exotoxin B (SpeB) proteolytically activates GSDMA, thereby triggering pyroptosis (Deng et al., 2022; LaRock et al., 2022). Recent research has revealed that the NS2B3 protease of the Zika virus specifically targets and cleaves GSDMD, resulting in pyroptosis in infected host cells (Yamaoka et al., 2021; Kao et al., 2023).

### NM-induced pyroptosis in specific cell types

Upon entering the human body, NMs, which are materials with a size of 1–100 nm, can interact with various cell types. The dynamics and outcomes of these interactions are highly dependent on the inherent properties of NMs, the nature of involved cells, and the specific microenvironment where the interaction occurs. Depending on these parameters, cells may respond in adaptive or defensive manners, which can lead to various outcomes, including cell death, specifically pyroptosis. Cell type plays a pivotal role in determining how cells interact with NMs and hence influences the potential induction of pyroptosis. Different cell types may elicit distinct responses to NM exposure because of their unique physiological features and functions. Some cells may be more susceptible to NM-induced pyroptosis based on their inherent biological characteristics. Current research on NM-induced cell pyroptosis primarily focuses on cancer treatment and covers NM-mediated magnetic hyperthermia, photothermal therapy, targeted tumor metabolism, chemotherapeutic drug delivery, and comprehensive therapies involving multiple strategies. These applications have been summarized in numerous reviews (Wu et al., 2021; Chen Y. Y. et al., 2022). In the current review, the regulation of pyroptosis by NMs in different types of non-tumor cells is emphasized and summarized (Table 1).

**TABLE 1:** Summary of NMs induce pyroptosis in non-tumor cells.

NM types	Cell types	Experimental subjects	Target	References
Silica nanoparticles	Macrophages	RAW264.7 cells	NLRP3/Caspase-1/GSDMD/IL-1β	Ma et al. (2022)
Multi-walled carbon nanotubes	Macrophages	THP-1 cells	NLRP3/Caspase-1/IL-18/IL-1β	Wang et al. (2020)
Silica nanoparticles	Macrophage	RAW-ASC cells	TLR4/NLRP3/NF-κB/Caspase-1/GSDMD/IL-1β	Yin et al. (2022)
Multi-walled carbon nanotubes	Macrophages	primary human macrophages	NLRP3/Caspase-1/GSDMD/IL-1β	Keshavan et al. (2021)
Indium-tin-oxide	Macrophages	Mouse peritoneal macrophages	NLRP3/Caspase-1/IL-1β	Naji et al. (2016)
Single-walled carbon-nanohorns	Macrophages	J774A.1 cells	Caspase-1/IL-1β	He et al. (2018)
silver nanoparticles	Macrophages	THP-1 cells	NLRP3/ASC/Caspase-1/IL-1β	Simard et al. (2015)
Iron oxide nanoparticles	Macrophages	bone marrow-derived macrophages	NLRP3/Caspase-1/GSDMD/IL-1β	Liu et al. (2018)
Boron nitride nanotubes	Macrophages	Mouse alveolar macrophages/THP-1 cells	NLRP3/Caspase-1/IL-18/IL-1β	Kodali et al. (2017)
Molybdenum trioxide nanoparticles	Macrophages	Mouse alveolar macrophages	NLRP3/ASC/Caspase-1/IL-1β	Huber and Cerreta (2022)
Zinc oxide nanoparticles	Hepatocytes	Rat hepatocytes/HepG2 cells	NLRP3/Caspase-1/GSDMD/IL-1β	Pei et al. (2023)
Silica nanoparticles	Hepatocytes	L02 cells	ROS/NLRP3/Caspase-1/IL-1β	Zhang et al. (2018)
CdSe/ZnS quantum dots	Hepatocytes	L02 cells	ROS/Ca^2+^/NLRP3	Lu et al. (2016)
Arsenic and polystyrene-nanoplastics	Hepatocytes	Mouse hepatocytes	NLRP3/ASC/Caspase-1/GSDMD	Zhong et al. (2022)
Polysaccharide nanofiber-stabilized pickering emulsion microparticles	Hepatocytes/Macrophages	HepG2 cells/KUP5 cells	Caspase-1/IL-1β	Li et al. (2023)
Sodium stabilized mesoporous aluminosilicate nanoparticles	Dendritic cells	DC2.4 cells	K^+^/Caspase-1/GSDMD/IL-1β	Tang et al. (2022)
Molybdenum disulfide quantum dots	Microglials	BV2 cells	NLRP3/Caspase-1/IL-1β	Yang et al. (2020)
Silica nanoparticles	Microglials	N9 cells	ROS/Caspase-1/GSDMD/IL-1β	Du et al. (2019)
CdTe and CdTe@ZnS quantum dots	Microglials	BV2 cells	ROS/NLRP3/NF-κB/Caspase-1/IL-1β	Liang et al. (2020)
Silica nanoparticles	Cardiomyocytes	AC16 cells	NLRP3/Caspase-1/GSDMD/IL-1β	Wang et al. (2022)
Hydroxyapatite nanoparticles	Vascular smooth muscle cells	A7R5 cells	Ca^2+^/ROS/NLRP3/Caspase-1/GSDMD	Xia et al. (2022)
Graphene oxide	Endothelial cells	human umbilical vein endothelial cells	NLRP3/GSDMD	Cao et al. (2021)
amine-polystyrene nanoplastics	Pulmonary epithelial cells	MLE-12 cells	Irg1/NF-κB/NLRP3/Caspase-1	Wu et al. (2023)
multi-walled carbon nanotubes	Lung fibroblasts	MRC-5 cells	NLRP3/Caspase-1/IL-1β	Hussain et al. (2014)
Coal dust nanoplastics	Pulmonary epithelial cells	A549 and BEAS-2B cells	Caspase-1	Zhang et al. (2022)
Zinc oxide nanoparticles	Keratinocytes	HaCaT cells	NLRP3/ASC/Caspase-1/GSDMD	Chen et al. (2022b)
Nanodiamond	Platelet	Mouse platelet	ROS/NLRP3/Caspase-1	Hung et al. (2022)

### Macrophages

Macrophages, which are phagocytic cells that participate in purging foreign substances, are abundantly present in all tissues in the body (Mantovani et al., 2004). As NMs represent exogenous entities, their entry into the system primarily triggers a clearance response from these macrophages. As such, macrophages are more susceptible to NM stimulation. The intensity and outcomes of this reaction are based on the interplay between macrophages and NMs; one such potential consequence is pyroptosis. Yin et al. found that silica-induced macrophage pyroptosis, which is an NLRP3-dependent process that requires TLR4 recognition and NF-κB mediation, is related to silica-induced lung inflammation and fibrosis (Yin et al., 2022). Reisetter et al. reported that macrophages exposed to long and rigid carbon nanotubes undergo pyroptosis, which is characterized by inflammasome activation, caspase-1 activation, and IL-1β release. The addition of caspase-1 and pyroptosis inhibitors reduces the cytotoxicity of carbon nanotubes (Keshavan et al., 2021). Some researchers have reported that certain NMs, such as hollow carbon spheres and graphene oxide, induce inflammasome activation, caspase-1 cleavage, and IL-1β release in mouse bone marrow-derived macrophages and human macrophage cell lines without causing cell death (Andón et al., 2017; Mukherjee et al., 2018). These studies have suggested that the occurrence of pyroptosis is closely related to NM type. Macrophages have a remarkable plasticity as potent phagocytic cells that can modify their functional phenotype in response to signals from their microenvironment. This plasticity explains the contrasting roles of macrophages at different inflammation stages. For example, they exhibit pro-inflammatory (M1) phenotypes during the reaction phase and anti-inflammatory (M2) phenotypes during resolution, a process known as macrophage polarization (Mantovani et al., 2002), which is influenced by NMs (Huang et al., 2018). However, the interplay between NM-induced macrophage pyroptosis and macrophage polarization remains unexplored.

### Dendritic cells (DCs)

Similar to macrophages, DCs possess various extracellular and intracellular receptors that can recognize different stimuli, including PAMPs and DAMPs. Because of the presence of these receptors, DCs can recognize and respond to NMs by triggering immune responses. Studies on NMs and DCs have focused on their application in nanomedicine, particularly cancer therapy, including research on manipulating immune responses against tumors and novel vaccine formulations. Tang et al. demonstrated that sodium-stabilized mesoporous alumino-silicate NMs induce pyroptosis in DCs by releasing a Na^+^ surge in a pH-responsive manner (Tang et al., 2022). This event subsequently triggers pro-inflammatory factor production, thereby amplifying antitumor immune responses. However, NM-induced pyroptosis in DCs has been rarely explored likely because of the inherent characteristics of DCs or limitations in cell models used; this gap is addressed in our discussion.

### Neutrophils

Neutrophils can eliminate invading foreign substances through various strategies, such as phagocytosis, degranulation, antimicrobial factor secretion, and neutrophil extracellular trap (NET) release. Silver nanoparticles (AgNPs) can rapidly penetrate neutrophils; thus, atypical cell death is induced, and IL-1β is released. This cell death is distinct from apoptosis and necrosis, and it can be reversed by caspase-1 and caspase-4 inhibitors. However, the specific mode of cell death has not been confirmed. Additionally, AgNPs can induce NET release (Liz et al., 2015). However, this process is not reversed by caspase inhibitors. By far, no definitive reports have described NM-induced neutrophil pyroptosis possibly because activated human neutrophils can digest carbon-based NMs in an MPO-dependent manner through NET release (Farrera et al., 2014). In fact, the resistance of neutrophils to pyroptosis is considered unique among inflammasome signaling cells.

### Hepatocytes

The liver is regarded as a critical target of NM toxicity. In addition to pyroptosis in macrophages, NM-induced cell pyroptosis in hepatic cells has been explored. Pei et al. found that ZnO NMs disrupt zinc homeostasis in rat livers and induce oxidative stress damage (Pei et al., 2023). These events trigger the assembly of the NLRP3-ASC-caspase-1 inflammasome and the activation of GSDMD; consequently, pyroptosis is stimulated, and pro-inflammatory cytokines such as IL-1β are released. Suppressing oxidative stress can protect against pyroptosis in liver cells exposed to ZnO NMs. Zhang et al. revealed that silica NMs cause hepatocyte cytotoxicity in a dose- and time-dependent manner; as a result, caspase-1-dependent pyroptosis occurs, but this process can be alleviated by reactive oxygen species (ROS) scavengers (Zhang et al., 2018). Lu et al. reported that quantum dots (QDs) induce the production of mitochondrial ROS (mtROS) in hepatocytes in a concentration-dependent manner; eventually, NLRP3 becomes activated, and pyroptosis takes place. mtROS and total ROS scavengers can mitigate QD-induced NLRP3 activation and pyroptosis (Lu et al., 2016). These studies have suggested that oxidative stress plays a pivotal role in NM-induced pyroptosis in hepatocytes.

### Other cells

In addition to immune cells and hepatocytes, other cell types, including bronchial epithelial cells, vascular smooth muscle cells, microglial cells, and keratinocytes, undergo NM-induced cell pyroptosis. For instance, Hussain et al. reported that multi-walled carbon nanotubes trigger NLRP3 inflammasome activation and consequent cell pyroptosis in primary human bronchial epithelial cells in a concentration- and time-dependent manner (Hussain et al., 2014). Similarly, Xia et al. found that hydroxyapatite nanoparticles disrupt Ca^2+^ homeostasis in vascular smooth muscle cells; subsequently, they cause mitochondrial dysfunction and cell pyroptosis (Xia et al., 2022). Consistent with previous studies on these NM-induced cellular changes, Du et al.’s study revealed how silica nanoparticles initiate mitochondrial ROS production, GSDMD cleavage, and pyroptosis in microglial cells (Du et al., 2019). Chen et al. demonstrated the combined effect of zinc oxide nanoparticles and UVB exposure, which can provoke NLRP3 inflammasome activation and pyroptosis in keratinocytes (Chen B. et al., 2022). These findings collectively highlight the potent influence of various NMs on different cell types.

### Properties of NM-induced pyroptosis

The morphological manifestations of NM-induced pyroptosis are consistent in various cell types, characterized by pre-rupture phenomena such as membrane bubbling, vesicular protrusions, swelling, and cell flattening. Mechanistically, NMs trigger the classical caspase-1-mediated pathway of pyroptosis in non-tumor cells, predominantly involving the GSDMD molecule, although some studies have not assessed the expression of GSDM proteins. The surprising uniformity of this pyroptosis mechanism in different studies suggests that different activation pathways may interact with the classical one, potentially overshadowing their presence. While the secretion of mature IL-1β has been detected in existing research, the absence of assays for the release of large proteins such as LDH indicates that these studies cannot distinguish between sublethal and lethal NM-induced pyroptotic cell death. Furthermore, *in vivo*, NM-induced pyroptosis can lead to a cascade of effects, including exacerbated local or systemic inflammatory responses attributed to cytokine release to tissue damage caused by cell rupture and cellular content leakage. Over time, these events may contribute to disease progression, fibrotic tissue formation impairing organ function, or initiation of repair processes that alter tissue integrity through scarring. Therefore, extensive toxicological studies should be performed to verify the long-term effects of various NMs by considering factors such as dose dependency and individual biological variability.

### Unexplored aspects and future perspectives

Despite extensive research on the potential of NMs to induce pyroptosis, remarkable knowledge gaps remain. Current studies primarily focus on how NMs exploit pyroptosis to combat tumors through various mechanisms, including direct cytotoxic effects on tumor cells and enhanced tumor immunogenicity. However, studies have yet to explore the effects of NMs on normal tissues and cells, such as various immune cells and drug metabolism-related cells (e.g., hepatocytes and renal cells). Specifically, studies should investigate whether NM-induced pyroptosis leads to potential nanotoxicity in these non-tumor cell environments. Further research should address this gap by investigating the interactions of NMs and healthy cells and their potential to trigger cell pyroptosis. Thus, any adverse effects of NMs can be assessed, and their safe application in clinical settings can be guaranteed.

To evaluate the pharmacodynamics and toxicology of NMs, researchers should develop and explore more reliable *in vitro* and *in vivo* models. Typically, studies on the pharmacological and toxicological characteristics of NMs are conducted in three scenarios: *in vitro* studies, studies on primary cells or transformed cells from human or animal sources, or studies using different animal models *in vivo*. However, *in vitro* and *in vivo* methods have inherent limitations that should be considered. For example, *in vitro* studies on DCs often involve stable cell lines derived from humans or mice, but these immortalized cell lines may not accurately replicate the characteristics of their primary cell lines. Therefore, the reliability of these results largely depends on experimental design parameters. Similarly, healthy animal models are used to evaluate the pharmacodynamics and toxicity of NMs. However, from an immunological perspective, healthy individuals and those with underlying diseases or aging may react differently to NMs. Therefore, the same NM may exhibit various therapeutic or toxic effects on different individuals even at the same dose. Furthermore, this variability may extend to the incidence of phenomena such as pyroptosis.

Current research on how NMs induce pyroptosis primarily uses a descriptive approach and focuses on observable phenomena associated with this process. The complex molecular mechanisms of pyroptosis, involving interactions between NMs and cells, are poorly understood and still in the early stages of research. Pyroptosis is a multistep process that involves various proteins and signaling pathways, such as inflammasomes, GSDMs, and caspases. However, studies have yet to clarify whether NMs directly interact with these molecules or they trigger upstream events leading to pyroptosis. Additionally, different NM types may induce pyroptosis through different mechanisms, thereby adding complexity to this field. Furthermore, some NMs show contradictory results although many NMs effectively induce pyroptosis. These variations may be attributed to different properties of NMs. Therefore, the effect of these properties on the induction of pyroptosis should be systematically studied.

Current studies on how NMs induce pyroptosis mainly focus on their composition, size, shape, and surface modifications. However, studies have not explored the influence of the biological environment on NMs. Once introduced into a biological system, NMs can rapidly interact with various biomolecules, including proteins, nucleic acids, lipids, and even metabolic byproducts, because they have a nanoscale size and a large surface area-to-volume ratio. Through this interaction, a NM–protein corona forms. The protein corona may regulate several aspects of NM behavior in the body, including cell uptake, response, accumulation, degradation, and clearance (Saptarshi et al., 2013). Furthermore, proteins adsorbed on a NM surface may undergo conformational changes; consequently, their immunogenicity changes, and the body’s immune homeostasis becomes disrupted. These interactions and subsequent modifications of NMs and biomolecules may considerably affect how NMs induce pyroptosis. Therefore, these complex interactions should be comprehensively understood to predict and control the cytotoxic effects of NMs in therapeutic applications.

The impact of environmental pollution on NM-induced pyroptosis has been largely overlooked. Before entering biological systems, environmental factors such as bacterial components or allergens can easily adsorb onto a NM surface, causing contamination. For example, certain bacterial components, such as LPS, cannot be easily removed completely even if NMs with attached bacteria are sterilized before an experiment. LPS is a heat-resistant and widely distributed bacterial component, which is a ubiquitous potential contaminant even in the absence of live bacteria. Current LPS detection methods are easily interfered by various factors, such as buffer components and detergents (Li et al., 2015; Schwarz et al., 2017); consequently, excluding LPS interference in NM experiments is difficult. The mechanisms by which LPS regulates immune responses are complex, and they may exacerbate inflammation or induce immune tolerance. Therefore, the presence of environmental contaminants such as LPS on NMs can remarkably affect the interaction of these materials with biological systems; potentially, they alter pyroptosis induction.

## Conclusion

This review comprehensively outlines the mechanisms and classifications of cellular pyroptosis. It also summarizes how various types of NMs can induce pyroptosis in nontumor cells. Nonetheless, further studies should explore other issues such as how NMs interact, how the characteristics of NMs influence pyroptosis, and how biological and environmental factors affect these processes. Future research will not only expand our understanding of the complex interactions between NMs and cellular systems but also provide a foundation for the safer and more effective use of nanotechnologies in clinical scenarios. As we further elucidate these complexities, we look forward to developing novel therapeutic strategies, thereby maximizing the potential of nanotechnology combined with cellular pyroptosis, which can be pivotal for medical advancements.
